# Transient Colonic Intussusception Versus Colon Adenocarcinoma: A Case Report on Ultrasound Features in the Emergency Department

**DOI:** 10.7759/cureus.30710

**Published:** 2022-10-26

**Authors:** Delange Augustin, Jonacé Gérald, Guy-Florent Lafontant, Olnick Joseph, Early Mushana Zidor

**Affiliations:** 1 Radiology, Hôpital de l'Université d'État d'Haïti, Port-au-Prince, HTI; 2 Surgery, Hôpital de l'Université d'État d'Haïti, Port-au-Prince, HTI; 3 Internal Medicine, Hôpital Universitaire de la Paix, Delmas, HTI

**Keywords:** colon adenocarcinoma, adult intussusception, transient colonic intussusception, pseudokidney sign, target sign

## Abstract

Intussusception in adults is a rare pathology due to the telescoping of a bowel segment into a section adjacent to it. Almost all cases are linked to a pathological lead point, which is often a colorectal carcinoma where the intussusception involves the large intestine. Likely to occur in the same clinical setting, the differential diagnosis between intussusception and colon carcinoma by ultrasound in the emergency department can be quite challenging. We present a rare case of transient colonic intussusception with a well-differentiated colon adenocarcinoma as the lead point in a 43-year-old patient. The point of care ultrasound (POCUS) revealed the target sign characteristic of intussusception at the level of maximum pain, associated with a pseudokidney sign. However, the pseudokidney sign was indeed an adenocarcinoma of the ascending colon visualized by colonoscopy and per op.

## Introduction

Intussusception is a condition in which part of the intestine folds into the section adjacent to it. It is an extremely rare entity, and hence its diagnosis in adults requires strong clinical suspicion. Challenges often arise because the signs and symptoms are nonspecific and frequently mimic many alternative diagnoses [1]. Unlike in children, almost 90% of cases of intussusception in adults have a pathological lead point (usually malignant in the large intestine and benign in the small intestine) [2,3,4].

Imaging is usually required to make the diagnosis, and fluoroscopy with contrast enema remains the gold standard. However, CT, being the modality of choice for the evaluation of acute abdomen in adults, has been reported to make more diagnoses of intussusception as well as transient intussusception, which is often an incidental finding. Abdominal ultrasound can also identify the characteristic target sign of intussusception in some cases, particularly in patients with a palpable abdominal mass that is more than 90% sensitive [2,4].

If intussusception is not diagnosed promptly, it puts the patient at risk for severe complications that can jeopardize the vital prognosis. Therefore an early and prompt diagnosis is crucial for the optimal management of the condition [2,5]. We present a case report where we highlight the sonographic features of intussusception in adults and the challenges of differential diagnosis with colon adenocarcinoma in acute settings.

## Case presentation

This case report concerns a 43-year-old woman who was seen at the emergency department for an acute exacerbation of intermittent abdominal cramping pain localized at the right lumbar region, which she had been experiencing for three years. A review of systems also revealed progressive weight loss. She reported having experienced an episode of melena for about a year. Physical examination revealed a soft, depressible abdomen with no evidence of a palpable mass. The results of the complete blood count revealed moderate normochromic normocytic anemia with a hemoglobin level of 10.6 g/dl.

**Figure 1:**
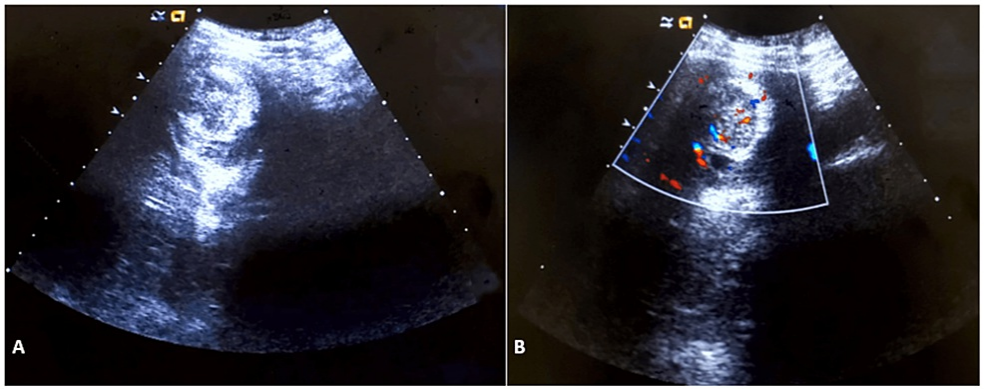
Intussusception - image 1 A. Cross-sectional 1-4 MHz grayscale ultrasound image obtained over the right lumbar region demonstrates the target sign (anteroposterior diameter of 5.41 cm). B. Target sign with Doppler

**Figure 2:**
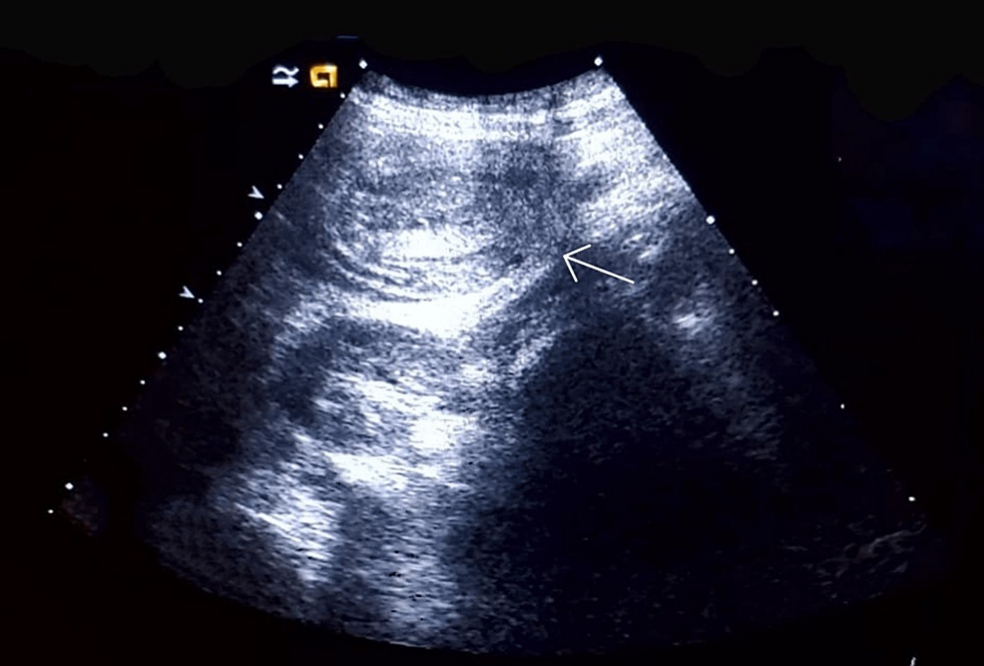
Long-axis 1-4 MHz ultrasound image obtained over the right lumbar region demonstrates the pseudokidney sign (long-axis diameter of 6.50 cm)

She was transferred to the radiology emergency department. There, an abdominal ultrasound revealed a target sign, located in the right lumbar region, in the area of maximum pain, with an anteroposterior diameter of 5.41 cm, associated with a pseudokidney sign at the ipsilateral side, measuring 6.50 cm on its long axis. This led to the diagnosis of right colonic intussusception (Figures 1-2). A colonoscopy was suggested. She refused to be hospitalized urgently despite receiving counseling and pain that had been bearable until then. She preferred to continue with the follow-up on an outpatient basis, without medical clearance while waiting to undergo the colonoscopy.

**Figure 3:**
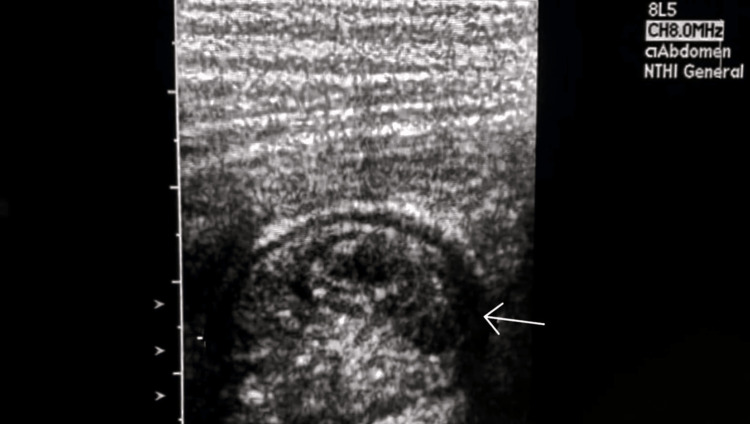
Intussusception - image 2 Cross-sectional 5-8 MHz ultrasound image obtained over the right lumbar region demonstrating the target sign

**Figure 4:**
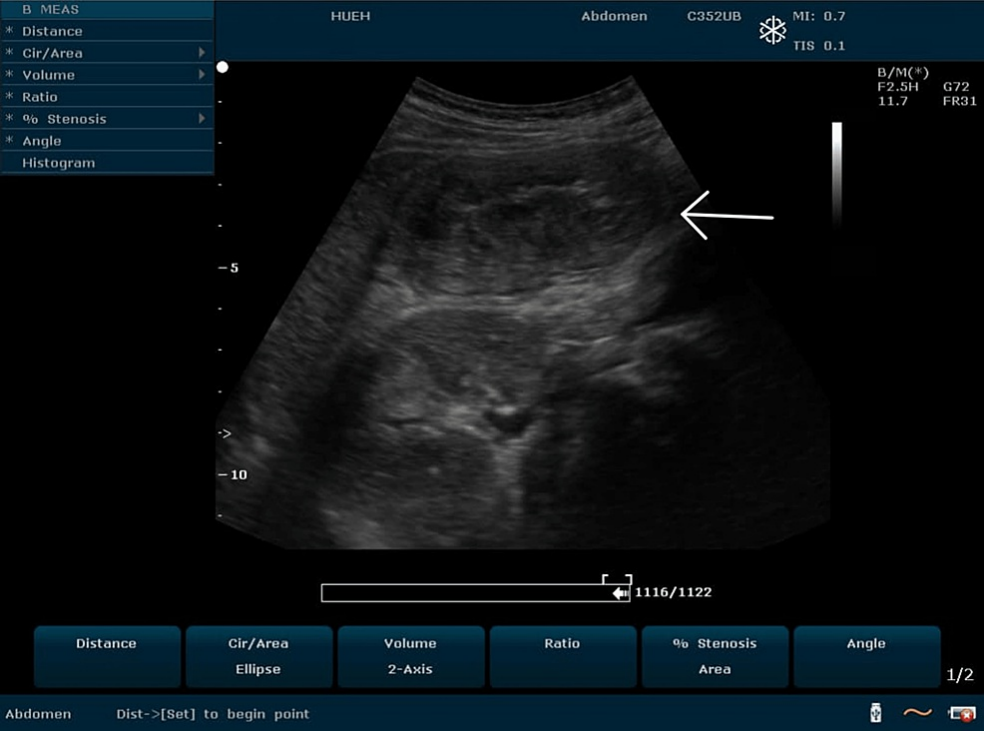
Intestinal mass Long-axis 2-6 MHz ultrasound image obtained over the right lumbar region showing a pseudokidney sign

Within a week, her clinical picture worsened with an intermittent increased abdominal pain that was accentuated in the right lumbar region, radiating towards the epigastric, associated with episodes of nausea and non-bilious vomiting. She was emergently hospitalized. The physical examination revealed a right lumbar rebound tenderness, with no evidence of a palpable mass. A bedside ultrasound was performed, which showed a target sign on the right lumbar region at the point of maximum pain, measuring 3.32 x 2.10 cm, and a pseudokidney sign measuring 6.81 x 3.13 cm located on the right flank (Figures 3-4). She was placed on painkillers and strictly monitored as they waited to complete the preoperative assessments for surgery the next day.

**Figure 5:**
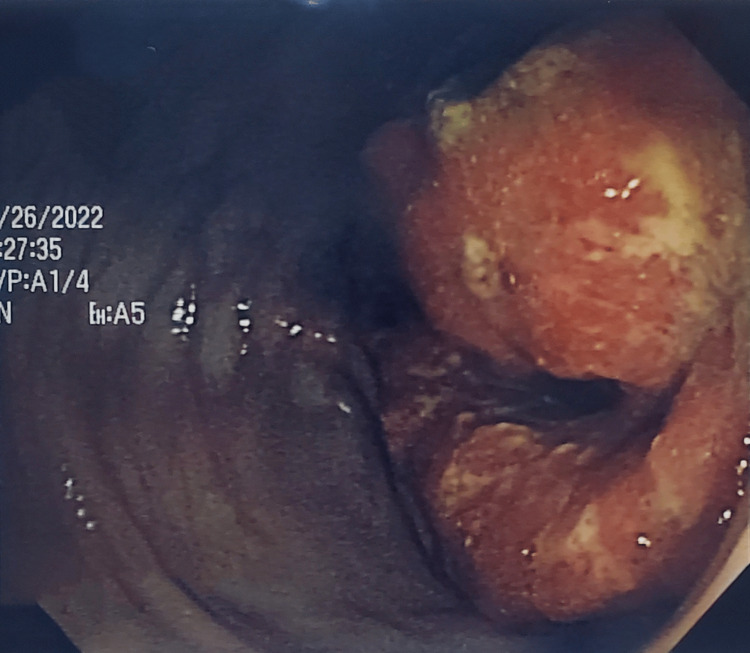
Adenocarcinoma of the colon: colonoscopy, the middle segment of the ascending colon

Due to a gradual improvement of her symptoms until a period of full pain resolution and being hemodynamically stable, the intervention was postponed until the results of the colonoscopy were obtained. The colonoscopy showed a vegetating lesion involving the entire circumference of the colon and 80% of its lumen at the level of the middle segment of the ascending colon, measuring 5-10 cm in length (Figure 5), with a normal cecum. Six biopsies were performed and they revealed a malignant glandular proliferation, showing irregular, disordered basophilic glands of various sizes, with a stratified coating and marked atypia. The tumor ulcerated the mucosal surface and infiltrated deeply. The stroma was very inflammatory. The colonic mucosa was also inflamed, and the areas near the tumor had dysplastic glands. These findings led to the diagnosis of a well-differentiated infiltrating adenocarcinoma.

**Figure 6:**
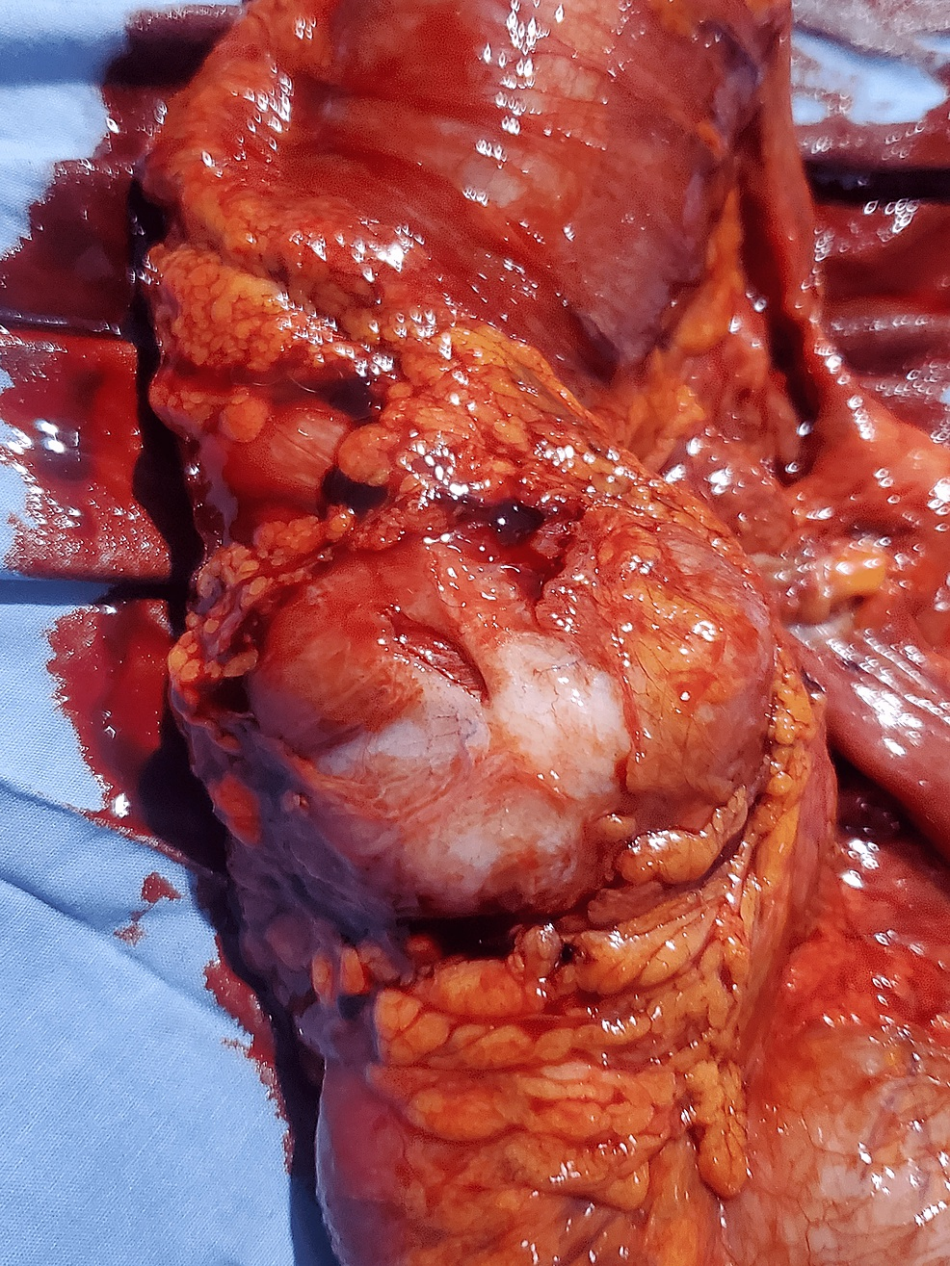
Colon adenocarcinoma: surgical specimen, external view

**Figure 7:**
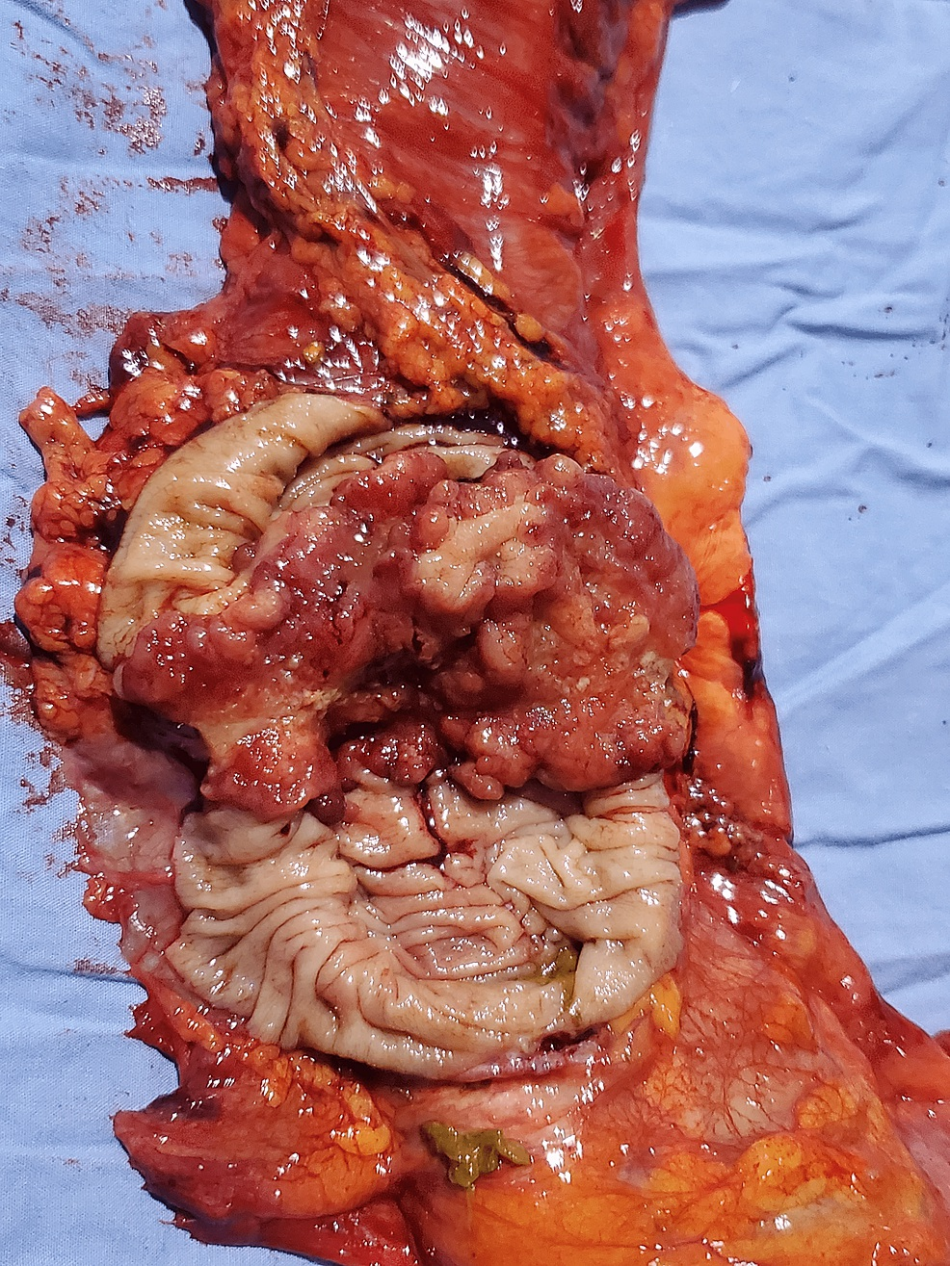
Adenocarcinoma of the colon: surgical specimen, intraluminal view

**Figure 8:**
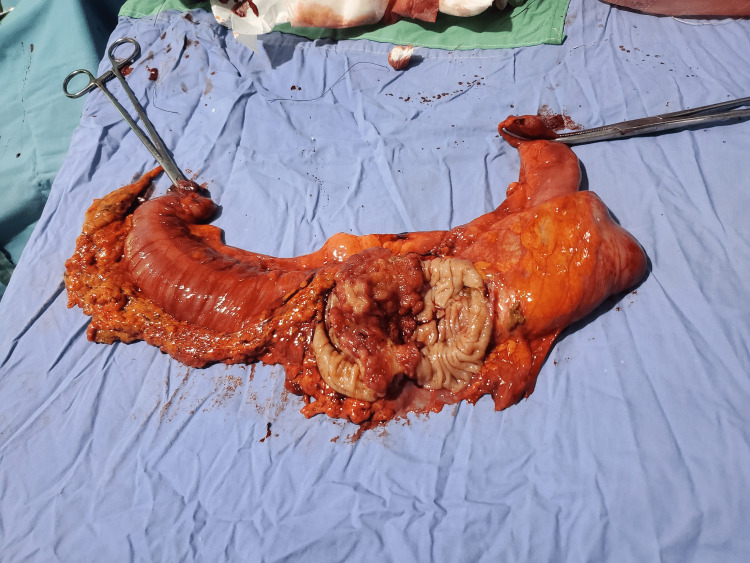
Right hemicolectomy: adenocarcinoma of the ascending colon

Preoperatively, a control ultrasound was performed during the asymptomatic phase, which highlighted a pseudokidney sign, but without evidence of a target sign. She then underwent a right hemicolectomy with the postoperative diagnosis of transient colonic intussusception on the adenocarcinoma of the ascending colon. The surgical specimen revealed a cauliflower-shaped mass 1/3 in the middle and 1/3 in the upper side of the ascending colon, associated with mesenteric lymphadenopathy and without any evidence of colonic intussusception, necrosis, or intestinal perforation (Figures 6-8). She was stable postoperatively and was referred to oncology for follow-up care.

## Discussion

Intussusception is extremely rare in adults and usually presents with intermittent abdominal pain, along with bloating, nausea, non-bilious vomiting, or bilious in case of obstructions. The classic triad of abdominal pain, vomiting, and passage of blood per rectum found in children is often absent in adults. In case of ischemia of the telescoped intestinal segment, it can present as episodes of the passage of blood through the anus. On physical examination, the pain may be diffused or localized with a reduction in intestinal transit. Without specific signs, imaging is generally necessary for the diagnosis, as the condition is associated with a plethora of alternative diagnoses [2,6,7].

The diagnosis of colonic intussusception in our case was discovered incidentally. It was characterized by visualization on the right lumbar area of a target sign with an anteroposterior diameter of 5.41 cm at the point of maximum pain and a pseudokidney sign on the ipsilateral side measuring 6.50 cm on the long-axis view. It has been reported that in an appropriate clinical setting, ultrasound can achieve 98.5-100% sensitivity in the diagnosis of intussusception. In general, the ultrasound signs used for making the diagnosis of intussusception are the target sign, the pseudokidney sign, and the crescent-in-doughnut sign [2,4]. On ultrasound, the pseudokidney sign presents as a kidney-shaped mass with a central hyperechoic region surrounded by a hypoechoic region [8]. However, it is not a pathognomonic sign. It was described for the first time in the context of colon carcinoma and has been found in a variety of gastrointestinal diseases [9,10].

Among other things, we have the target sign with a sensitivity of more than 90% in the presence of a palpable mass [2], and the crescent-in-doughnut sign [4]. In the presence of these signs, an anteroposterior diameter of more than 2 cm usually indicates ileocolic intussusception rather than a small bowel intussusception, but this has been observed mainly in children [11]. In terms of diameter, as the condition is rare in adults, no ultrasound data is available to date for the differential diagnosis between a colonic intussusception and a small bowel intussusception. However, as shown in our case, a target sign with an anteroposterior diameter of 5.41 cm located at the level of the right lumbar region, projecting to the ascending colon is much more likely to be a sign of colonic intussusception, rather than a small bowel intussusception.

One of the widely used radiological modalities for the diagnosis of intussusception in adults is the abdominal CT scan with a sensitivity of 58-100% and a specificity of 57-71% [12]. The presentation of intussusception on CT depends on the imaging plane and where along the bowel the images are taken. The most well-known is the so-called bowel-within-bowel configuration, in which the layers of the bowel are duplicated, forming concentric rings (CT equivalent of the ultrasonographic target sign) when imaged at angles to the right of the lumen, and a soft tissue sausage when imaged longitudinally [4].

Fluoroscopy with contrast enema remains the gold standard for the diagnosis of intussusception, which demonstrates the intussusception as an occluding mass prolapsing into the lumen, giving the “coiled spring” appearance [4]. However, usually, an acute-setting CT scan and point-of-care ultrasound (POCUS) are the main imaging modalities employed to evaluate an acute abdomen [2,4,13].

In a 2020 systematic review on the diagnostic accuracy of intussusception in children in the emergency department, POCUS had a sensitivity rate of 94.9% and a specificity rate of 99.1%. The main limitation of this study was the fact that the ultrasound was operator-dependent. The two main ultrasound signs used to make the diagnosis were the target sign and the pseudokidney sign [13]. Even if the target sign can also be found in cases of colon carcinoma and other inflammatory bowel pathologies, it is more sensitive and more specific for intussusception in an acute setting. In a previous report, the sonographic criteria of colon carcinoma were not defined precisely. More specific and precise sonographic indicators of colon carcinoma can be found with the aid of a high-frequency transducer (7.5 MHz) [13,14], which was also used in our case, highlighting the perfect target sign of intussusception associated with the clinical picture of nausea, non-bilious vomiting, and acute abdominal pain with rebound tenderness. The target sign was present through all the painful symptomatic phases in the zone of maximum pain and absent during the asymptomatic phase. The pseudokidney sign, present in the symptomatic phases as well the asymptomatic phase, was in fact the colon adenocarcinoma visualized per op.

The most common acute surgical complications of colon cancer are perforation, obstruction, and bleeding [15]. These differential diagnoses have been eliminated clinically, ultrasonographically, and per op. With regard to intussusception, diagnostic delays, and the risks of presentation in a clinical picture of severe complications such as occlusion, and ischemia of the telescoped intestinal segment, which can present through episodes of passages of blood per rectum, necrosis, and perforation, these presentations are part of the most common presentations of the acute complications of colon cancer [2,5,15] and maybe the reason for the underdiagnosis of adult intussusception in the context of colon cancer.

Given that nearly 90% of cases of intussusception in adults are related to a pathological lead point, and that 65% of them are associated with a gastrointestinal carcinoma, mostly colorectal carcinoma [16], any patient with suspected colon cancer who is seen at the emergency department for acute abdominal pain and presents with a target sign and a pseudokidney sign on abdominal ultrasound should be considered a case of intussusception until proven otherwise. In this case, the target sign, synchronous with the symptomatic phases, was the only characteristic ultrasound finding that helped in the differential diagnosis between colon adenocarcinoma and intussusception. The pseudokidney sign was, in fact, the adenocarcinoma of the ascending colon and served as a lead point for the transient colonic intussusception characterizing the acute symptomatic phase of the patient.

Percutaneous ultrasound-guided biopsy is a widely accepted procedure for the diagnosis of abdominal and retroperitoneal lesions, with high accuracy, low complication rates, and very low mortality rates [17-19]. In a retrospective study on the use of ultrasound-guided percutaneous biopsy, the diagnosis of gastrointestinal lesions was correct in 113/114 cases, with an accuracy rate of 99% [20]. We had the chance to perform the colonoscopy during the asymptomatic phase before surgery. However, in case of strong suspicion of intussusception, with the pseudokidney sign as a potential lead point, percutaneous ultrasound-guided biopsy on the pseudokidney sign mass could be an essential asset, not only to determine the nature of the mass but also to speed up the management time in case this mass is indeed the lead point of a potential intussusception.

## Conclusions

The ultrasound-based differential diagnosis between colonic intussusception and colonic adenocarcinoma can be quite difficult as both of them can present the same sonographic features. However, as our case report highlighted, this patient's condition raised strong suspicion related to colon cancer. She presented to the emergency room with nausea, vomiting, acute abdominal pain, and rebound tenderness at the right lumbar region associated with a target sign in every painful episode, which was in fact a case of transient colonic intussusception on a right colon adenocarcinoma. The pseudokidney sign, which was present even during the asymptomatic phase, was in fact the colon adenocarcinoma serving as the lead point for the intussusception. In an acute setting, any patient whose condition raises strong suspicion of colon cancer, presenting with a target sign and a pseudokidney sign during POCUS, should be considered as a case of intussusception until proven otherwise. The target sign was the key sonographic finding that allowed for the differential diagnosis between intussusception and colon carcinoma. Once the potential lead point is identified, an emergency percutaneous echo-guided biopsy can be performed to accelerate the delay of treatment, and thereby reduce the risk of severe complications. However, besides those concerning stable patients, more studies need to be conducted on the risks associated with the use of percutaneous ultrasound-guided biopsy in acute care units.
